# *Rht8* gene as an alternate dwarfing gene in elite Indian spring wheat cultivars

**DOI:** 10.1371/journal.pone.0199330

**Published:** 2018-06-21

**Authors:** Gomti Grover, Achla Sharma, Harsimar S. Gill, Puja Srivastava, N. S. Bains

**Affiliations:** Department of Plant Breeding and Genetics, Punjab Agricultural University, Ludhiana, Punjab, India; Institute of Genetics and Developmental Biology Chinese Academy of Sciences, CHINA

## Abstract

Optimizing wheat height to maximize yield has been an important aspect which is evident from a successful example of green revolution. Dwarfing genes (*Rht*) are known for yield gains due to lodging resistance and partitioning of assimilates into ear. The available and commercially exploited sources of dwarfism in Indian spring wheat are *Rht1* and *Rht*2 genes inspite of availability of over 20 dwarfing genes. *Rht8* a Gibberellic acid sensitive dwarfing gene is another reduced height gene commercially exploited in some Mediterranean countries. Two F_2_ populations segregating for *Rht1* and *Rht8* genes with each comprising 398 and 379 plants were developed by crossing European winter wheat cultivars Beauchamp and Capitole with Indian spring wheat cultivar PBW 621. Different genotypic combinations for *Rht1* and *Rht8* genes were selected from these populations through linked molecular markers and selected F_3:4_ lines were evaluated for various agronomic traits in a replicated trial. Reduction in plant height with *Rht8* and *Rht1* averaged 2.86% and 13.3% respectively as compared to the group of lines lacking dwarfing gene. Reduction was spread along all the internodes of wheat culm and reduction was lower as progress towards the lower internode. Grain number per spike and highest yield was observed in lines carrying only *Rht1* gene. Reduction in plant biomass was observed with either of the dwarfing gene. Longest coleoptile length and seedling shoot length averaged 4.4 ± 0.09 cm and 19.5 ± 0.48, respectively was observed in lines lacking any of the dwarfing gene. Negligible reduction of 6.75% and 2.84% in coleoptile length and seedling shoot length, respectively was observed in lines carrying only *Rht8* gene whereas F_3:4_ lines with *Rht1* gene showed 21.64% and 23.35% reduction in coleoptile length and seedling shoot length, respectively. Additive effect of genes was observed as double dwarfs showed 43.31% and 43.34% reduction in coleoptile length and seedling shoot length.

## Introduction

To meet the escalating demand of fast growing human population, there is need of continuous increase in wheat grain production. Optimizing plant stature to enhance productivity under ever changing and unpredictable climate is one of the major strategy of plant breeders. Reduced height in wheat is often associated with increase in yield, probably, owing to reduced lodging and enhanced partitioning of assimilates to the grain [1]. The control of plant height in wheat is known to be complex because of its polygenic nature and environmental effects. Genes with this ability have been difficult to detect, resulting in the vast majority of the world’s semi-dwarf wheat crop cultivars having their height reduction determined primarily by one of two major genes derived from the old Japanese cultivar ‘Norin 10’ [2]. Worldwide, during the past century, the average height of bread wheat (*Triticum aestivum* 2n = 6x = 42) has been dramatically reduced. For example, the average heights of wheat varieties have dropped from 150 cm to 90 cm in UK [3] and from 107 cm to the current height of around 90 cm in China [4]. In India, the Indo-Gangetic fertile crescent is the main wheat producing region and is classified as North Western Plain Zone (NWPZ) of India. Plant height in NWPZ has been steadily reduced from over 130 cm (tall traditional cultivars of pre-dwarfing era) to 84 cm (present day cultivars). The saga of green revolution in India is very well known and semi-dwarf varieties played a role of fuel for green revolution. *Rht1* and *Rht2* dwarfing genes were transferred from Japan to USA, USA to CIMMYT, Mexico and from Mexico to all over the wheat cultivated area through Japanese variety Norin-10. *Rht8* is mainly commercialized in Europe and transferred through Japanese variety Akakomugi from Japan to Italy and then from Italy to other parts of Europe [5]. These reduced height genes are effective in reducing plant height and have been widely adopted in wheat breeding programs since their introduction in the 1950s [6]. Their use has been associated with increased wheat yields particularly where conditions for growth are favorable [7]. *Rht1-B1b* and *Rht1-D1b* dwarfing alleles of *Rht1* and *Rht2* not only reduce the plant stature, but also reduce the coleoptile length which is the deciding factor for early emergence under water deficit or stress conditions and hence, effect the crop stand. It has also been well documented that dwarfing genes *Rht-B1b* and *Rht-D1b* are associated with Type I susceptibility to Fusarium head blight in wheat and low anther extrusion [8]. Numerous studies on *Rht* genes have concluded that *Rht8* gene reduces the plant height but it has negligible effect on coleoptile length [9, 10, 11]. The study on effect of combination of GA responsive *Rht8* gene and GA insensitive *Rht1* and *Rht2* genes have been conducted in Australian cultivars [11] and in some winter wheat cultivars. But the potential of combining these genes in Indian spring wheat background have not been previously assessed. Considering this, the aim of the study was to assess the potential of *Rht8* gene in combination with *Rht1* gene in elite spring wheat background under Indian environmental conditions.

## Materials and methods

### Population development

Two crosses (Beauchamp x PBW 621 and Capitole x PBW 621) were generated between cultivars Beauchamp and Capitole (having gene *Rht8c)* and genotype PBW 621 (having gene *Rht1)* in main season 2012–13. Beauchamp and Capitole are winter wheat varieties released in France in 1978 and 1964, respectively. These lines were selected from a diverse European winter wheat germplasm set of 376 cultivars that had been procured by Punjab Agricultural University, Ludhiana, Punjab, India from National Institute of Agricultural Botany, Cambridge, UK in 2010. PBW 621 is a spring wheat cultivar released in Indian in 2011 and widely grown in NWPZ of India. The F_1_ plants of these crosses were self-pollinated to generate F_2_ population. In wheat growing season 2014–15, 398 F_2_ plants from cross Beauchamp x PBW 621 and 379 F_2_ plants from Capitole x PBW 621 were space planted and plant height data was recorded. Further without any selection all of the F_2_ plants were allowed to self-pollinate to generate F_3_ progenies. The presence and absence of dwarfing allele(s) of both the *Rht1* and *Rht8* loci were determined by using linked molecular markers [12,13]. There is a substitution of one nucleotide in *Rht1* locus which leads to evolution of *Rht-B1b* allele (dwarfing allele) [14], hence, due to the difference of one nucleotide in the primer sequence, mis-annealing of primers was observed. Therefore, based on the plant height data and molecular data 16 and 17 F_3_ progenies of Beauchamp x PBW 621 and Capitole x PBW 621 cross, respectively were identified as being double dwarfs (*Rht1* + *Rht8*) or single dwarf (either *Rht1* or *Rht8*) or tall (no dwarfing allele). Two plants from each of these progenies were harvested separately and multiplied at Keylong, Himachal Pradesh (off-season research station of Punjab Agricultural University) and 45 of these F_3:4_ lines with similar growth period were selected for agronomic evaluation in main season 2016–17 at experimental area, Punjab Agricultural University, Ludhiana.

### Genotyping for *Rht1* and *Rht8*

Young leaves of parental lines and F_3_ progenies which seemed phenotypically homozygous were collected and genomic DNA was extracted using the standard CTAB (Cetyl trimethyl ammonium bromide) procedure [15]. The SSR marker WMS 261 for *Rht8* and gene specific dominant markers BF, WR1 and MR1 for *Rht1* were used. The in-vitro amplification (PCR) and PCR product were resolved as per the protocol described [12,13]. During the period of this study KASP assay was reported for *Rht1* gene [16]. KASP is a high throughput SNP based fluorescent genotyping technology. So, genotype of selected 45 F_3:4_ lines were also confirmed with KASP markers and genotypic composition of these groups are: both the dwarfing genes absent (*r*_*1*_*r*_*1*_*r*_*8*_*r*_*8*_), Rht1 absent and *Rht8* present in homozygous form (*r*_*1*_*r*_*1*_*R*_*8*_*R*_*8*_), *Rht1* in heterozygous form and *Rht8* absent (*R*_*1*_*r*_*1*_*r*_*8*_*r*_*8*_), *Rht1* in heterozygous and *Rht8* present in homozygous condition (*R*_*1*_*r*_*1*_*R*_*8*_*R*_*8*_), *Rht1* in homozygous form and *Rht8* absent (*R*_*1*_*R*_*1*_*r*_*8*_*r*_*8*_) and both *Rht1* and *Rht8* in homozygous form (*R*_*1*_*R*_*1*_*R*_*8*_*R*_*8*_).

### Coleoptile length and seedling shoot length assessment

For seedling traits, 12 seeds of each of the F_3:4_ line with no physical damage and of uniform size were placed in the middle of moist germination paper. The seeds were first sterilized with the fungicide to avoid any infection and placed in wet paper with germ end down and then paper was rolled as a ‘cigar’. The cigars were then placed vertically into a container with 5–7cm of water at the bottom and then the container was placed in an incubator at 20°C for 10 days under dark conditions. After 10 days, cigars were unrolled and average coleoptile length of seedlings was recorded from the base of the seed to the coleoptile tip. Total shoot length of seedlings was also measured.

### Agronomic evaluation

In 2014–15, F_2_ individuals were space planted and plant height was recorded. During main season 2015–16, F_3_ progenies were planted as plant to progeny row. Selected F_3_ plants were harvested individually and planted at offseason 2016 for multiplication to conduct a replicated trial in coming main season. The experiment for certain agronomic traits was conducted for selected F_3:4_ lines in main growing season 2016–17. These lines along with parents were planted as 7 x 7 square lattice design with three replications. Three plants from internal rows of each plot were randomly selected for observations to eliminate the border effect. Final plant height and individual internodal length was measured in centimeter at plant maturity. Ear length was also recorded. The internode below the spike was designated as peduncle length and successive to peduncle length was defined as first internode, second internode and so on. Days to flowering, total tillers per meter row, spikelet per spike, grain per spike, grain yield per plot, 1000-grain weight, plant biomass and harvest index were also recorded.

### Data analysis

Adjusted mean of each of the F_3:4_ line was calculated with the Analysis of variance of square lattice design using SAS ver. 9.3. The relative effect of different gene combinations was compared as a percent change with respect to tall group.

$$\%\;change\;with\;dwarfing\;gene\left(s\right)=\frac{Mean_{dwarf}-Mean_{tall}}{Mean_{tall}}\;x\;100$$

## Results

### Molecular marker assay for *Rht1* and *Rht8*

PCR assay of parental genotypes for *Rht1*, *Rht2* and *Rht8* confirmed that PBW 621 carries dwarfing allele of *Rht1* and both winter wheat parents Beauchamp and Capitole carry dwarfing allele of *Rht8* gene. Genotyping with WMS 261 marker showed a 192 bp band in Beauchamp and Capitole and approximately 165 bp band in PBW 621. Considering Norin-10, the source of dwarfism in Indian cultivar which carries both *Rht1* and *Rht2* gene [17], parents were screened for both *Rht1* and *Rht2*. Genotypes C-306 and C-518, the tall traditional varieties, widely grown before green revolution were used as a control. None of the parental genotype showed the presence of dwarfing allele of *Rht2* gene with DF-WR2 primer combination. With BF-MR1 primer combination for *Rht-B1a* allele (tall allele), all of the lines showed amplification but C-varieties and European winter wheat lines showed very high intensity PCR product whereas PBW 621 gave very faint band. For *Rht-B1b* allele (dwarf allele) BF-MR1 primer combination was used and faint band was obtained for European winter wheat lines and C-varieties, whereas comparatively intense amplified product was obtained for PBW 621. From these results, it has been concluded that *Rht1* gene is responsible for semi-dwarfing nature of spring wheat line used (PBW 621).

F_3_ progenies which seemed homozygous for plant height at early stages were chosen for molecular marker analysis for *Rht1* and *Rht8* genes. Leaves from all the plants of a progeny were bulked for DNA extraction so that a particular sample represent F_2_ genotype. For Beauchamp x PBW 621 cross, 85 F_3_ progenies were selected for molecular marker analysis. Out of 85, 19 progenies were observed to be positive for *Rht8* gene, 49 were heterozygous and 16 progenies were negative for *Rht8* gene with WMS 261 marker. Similarly, for cross Capitole x PBW 621, 75 progenies were selected for molecular marker analysis based on the phenotype of the F_3_ progeny. Among these 75 progenies, 15 were come to be positive, 41 were heterozygous and 15 were observed to be negative for Rht8 gene as revealed by marker WMS 261.

Based on the molecular marker data and phenotype data, 45 F_3:4_ lines with similar growth period were selected for agronomic and seedling evaluation. Because of the uncertainty about *Rht1* gene in the segregants, these 45 lines were genotyped with the KASP markers reported [16]. KASP based genotyping for *Rht1* revealed that out of 45, 12 segregants were heterozygous for *Rht1*. So, based on the genotyping of selected F_3:4_ lines instead of four homozygous groups, these lines were categorized into six different genotypic classes i.e. both the dwarfing genes absent (*r*_*1*_*r*_*1*_*r*_*8*_*r*_*8*_), *Rht1* absent and *Rht8* present in homozygous form (*r*_*1*_*r*_*1*_*R*_*8*_*R*_*8*_), *Rht1* in heterozygous form and *Rht8* absent (*R*_*1*_*r*_*1*_*r*_*8*_*r*_*8*_), *Rht1* in heterozygous and *Rht8* present in homozygous condition (*R*_*1*_*r*_*1*_*R*_*8*_*R*_*8*_), *Rht1* in homozygous form and *Rht8* absent (*R*_*1*_*R*_*1*_*r*_*8*_*r*_*8*_) and both *Rht1* and *Rht8* in homozygous form (*R*_*1*_*R*_*1*_*R*_*8*_*R*_*8*_).

### Effect on coleoptile length and seedling shoot length

**Fig 1:**
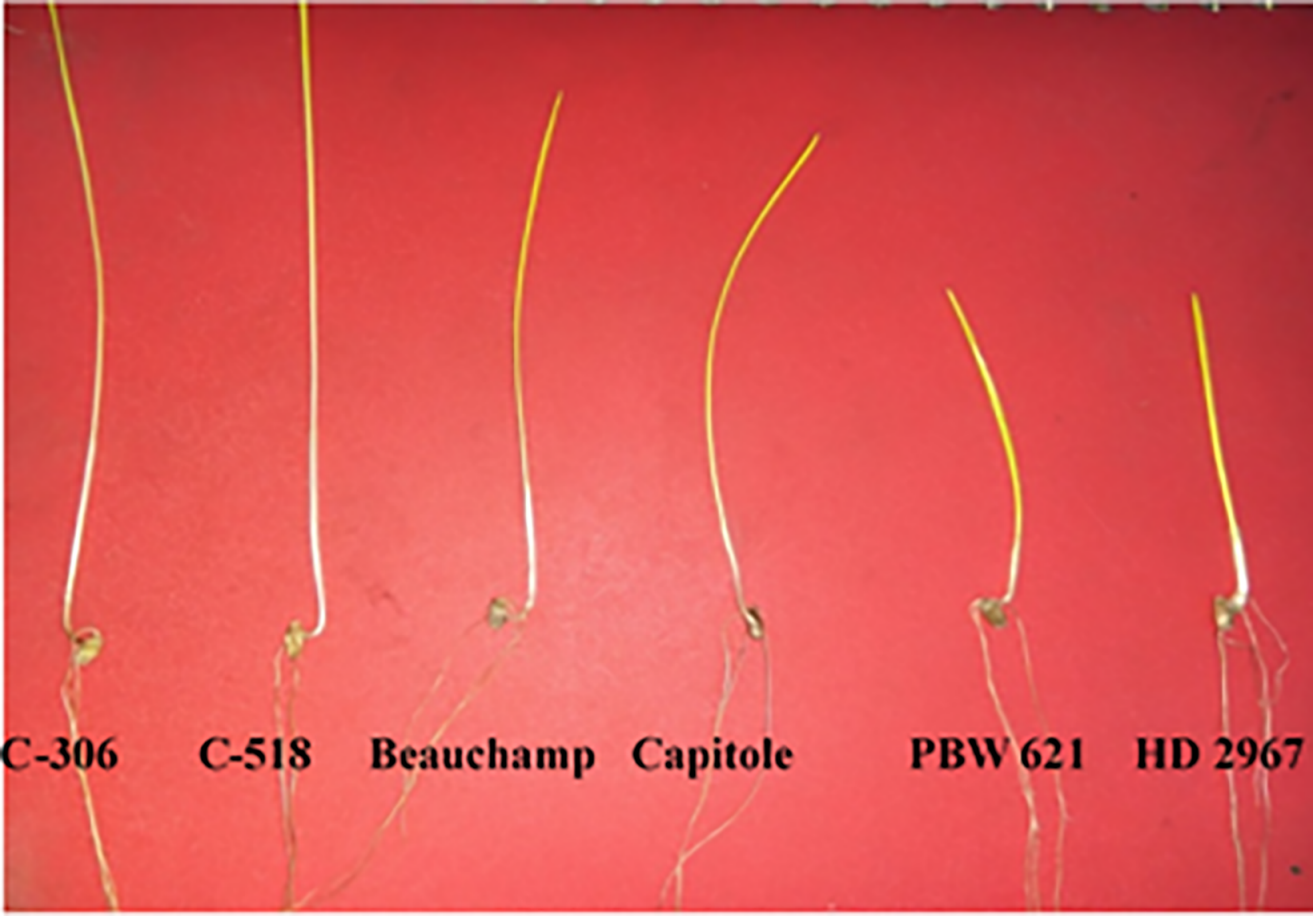
CL and SL of some cultivars.

**Fig 2:**
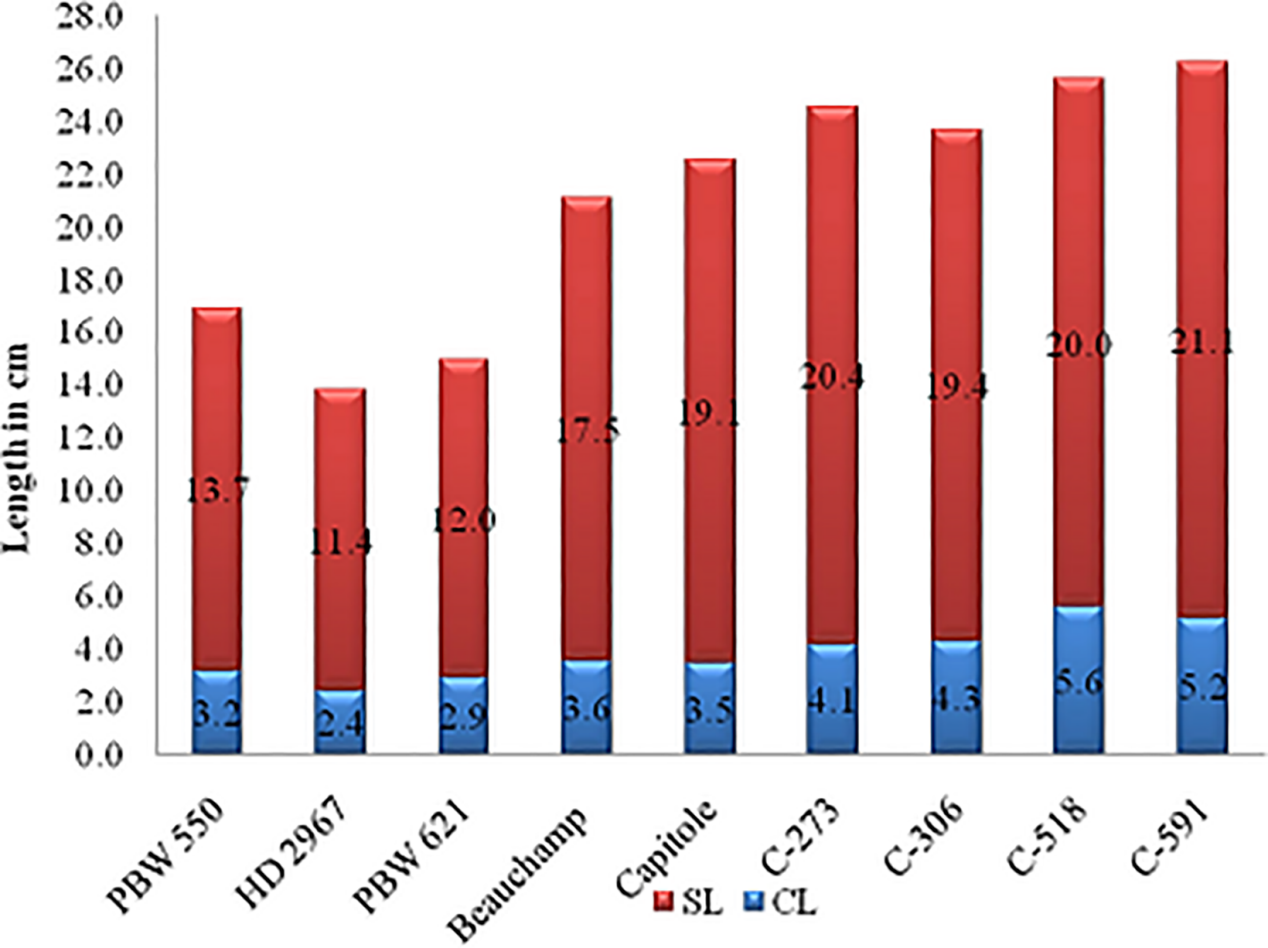
Graphical representation of CL and SL of some cultivars.

Coleoptile length (CL) of European wheat was marginally higher as compared to spring wheat cultivar PBW 621 whereas significant difference for seedling shoot length (SL) was observed between the European winter wheat lines and PBW 621. 3.6 cm and 3.5 cm of CL was recorded for Beauchamp and Capitole, respectively whereas PBW 621 was having 2.9 cm of CL. Total seedling shoot length for PBW 621 was 14.9 cm whereas for Beauchamp and Capitole it was 21.1 cm and 22.6 cm, respectively (Figs 1 and 2).

**Fig 3:**
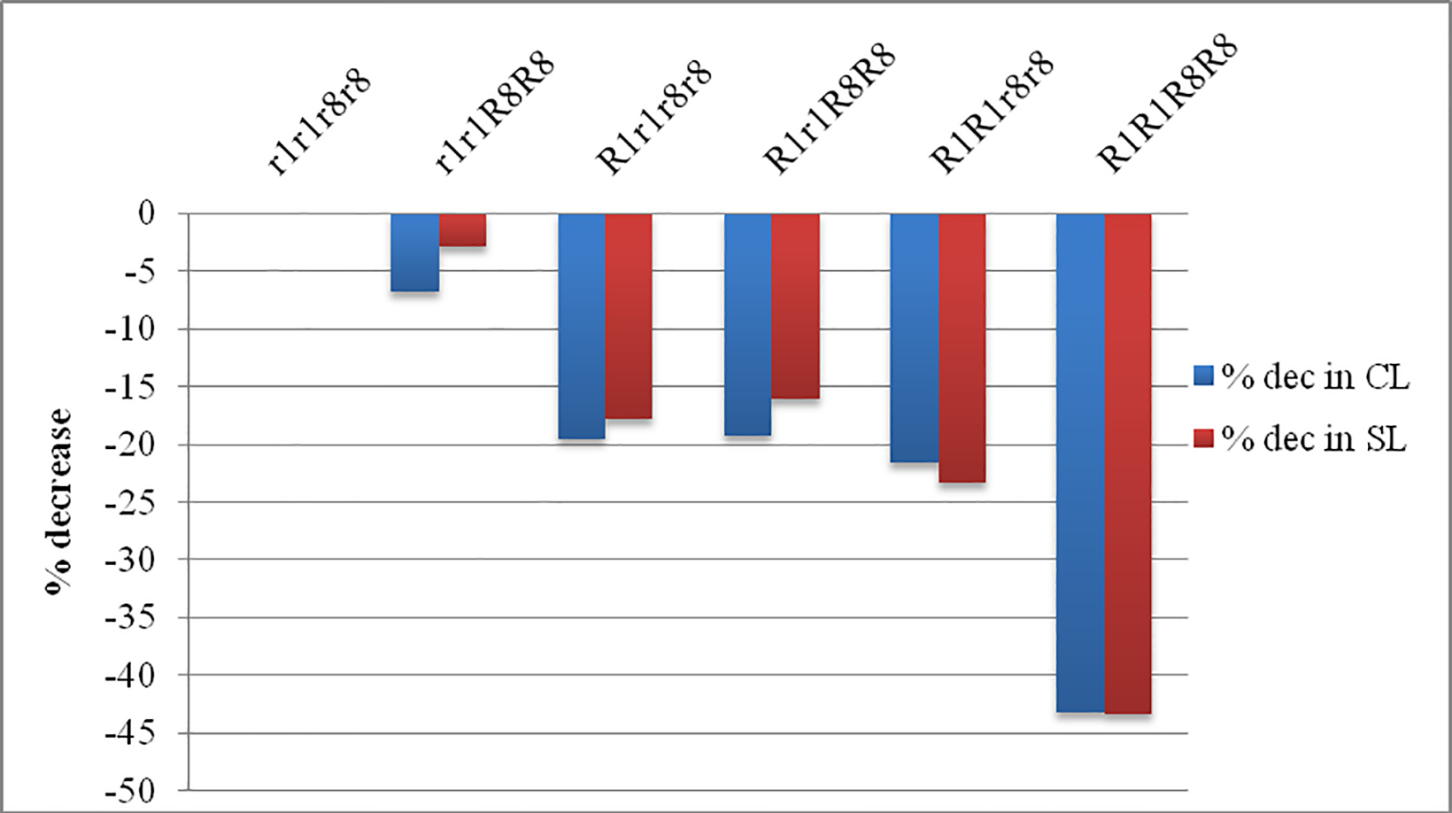
Percent decrease in CL and SL in each genotypic category.

Among different gene combinations of F_3:4_ lines, group *r*_*1*_*r*_*1*_*r*_*8*_*r*_*8*_, with no dwarfing genes were observed to have the longest CL and SL with mean value of 4.4 ± 0.09 cm and 19.5 ± 0.48 cm for CL and SL respectively. An average CL and SL of the plants which carry only *Rht8* dwarfing gene was 4.1 cm and 18.9 cm, respectively. Mean CL of both *R*_*1*_*r*_*1*_*r*_*8*_*r*_*8*_ and *R*_*1*_*r*_*1*_*R*_*8*_*R*_*8*_ category was 3.5 cm and for *R*_*1*_*R*_*1*_*r*_*8*_*r*_*8*_ it was 3.4 cm whereas mean SL for these three categories was 16 cm, 16.4 cm and 14.9 cm, respectively (Table 1). *Rht1* gene when present alone reduced the CL and SL by 21.64% and 23.35% and *Rht8* alone reduced these traits by 6.75% and 2.84% but when both of these genes were present in homozygous condition, reduction in CL and SL was observed to by 43.31% and 43.34%. Percent decrease in CL and SL of different classes as compare to tall group is shown in Fig 3.

**Table 1:** Effect of *Rht1* and *Rht8* on seedling traits (CL and SL).

Classes	Genotypic Classes	Coleoptile length			Seedling shoot length		
		CL (cm)	Range (cm)	% decrease in CL	SL (cm)	Range (cm)	% decrease in SL
No dwarfing gene detected	*r*_*1*_*r*_*1*_*r*_*8*_*r*_*8*_	4.4 ± 0.09	3.5–4.8	0	19.5 ± 0.48	15.6–21.9	0
*Rht8* present	*r*_*1*_*r*_*1*_*R*_*8*_*R*_*8*_	4.1 ± 0.10	3.6–4.6	-6.75	18.9 ± 0.42	17–20.9	-2.84
*Rht1* in heterozygous form	*R*_*1*_*r*_*1*_*r*_*8*_*r*_*8*_	3.5 ± 0.17	3.1–4.2	-19.51	16.0 ± 0.52	14.1–18.3	-17.77
*Rht1* in heterozygous form + *Rht8*	*R*_*1*_*r*_*1*_*R*_*8*_*R*_*8*_	3.5 ± 0.08	3.2–3.8	-19.33	16.4 ± 0.53	14–17.6	-16.03
*Rht1* in homozygous form	*R*_*1*_*R*_*1*_*r*_*8*_*r*_*8*_	3.4 ± 0.07	3.2–3.8	-21.64	14.9 ± 0.20	14.4–16	-23.35
*Rht1* + *Rht8*	*R*_*1*_*R*_*1*_*R*_*8*_*R*_*8*_	2.5 ± 0.10	2.2–2.8	-43.31	11.0 ± 0.55	9.3–12.1	-43.34

### Effect on plant height

Although parental lines are having different dwarfing genes and habit type (winter and spring) yet no significant variation between plant height of these lines were observed. Parental genotypes i.e. Beauchamp, Capitole and PBW 621 used in the study showed comparable plant height averaging 92 cm, 103 cm and 100 cm respectively. Whereas two populations developed from these parents were highly segregating for plant height. Wild type genotypic class (having no dwarfing gene) was taller among all with average plant height of 119.7 cm while both the dwarfing genes caused reduction in plant height with varying extent. Mean plant height of the lines with *Rht8* gene was 3 cm shorter (2.86%) than the tall category. *Rht1* caused approximately 13.3% reduction in plant height when present in homozygous form which indicated that extent of height reduction was stronger with *Rht1* as compare to *Rht8*. However, average plant height of lines with *Rht1* in heterozygous form was 8.7 cm taller than the lines which carry *Rht1* in homozygous form, suggesting the partial dominance nature of *Rht1* gene (Table 2).

**Table 2:** Effect of *Rht1* and *Rht8* on different agronomic traits.

Classes	Genotypic Classes	Plant Height (cm)	Spikelet number	Grain number/spike	1000-grain weight (g)	Yield (Kg/plot)	Plant Biomass (Kg/plot)	Harvest index
No dwarfing gene detected	*r*_*1*_*r*_*1*_*r*_*8*_*r*_*8*_	119.7 ± 1.75	21	52	36.9	1.3	4.782	0.278
*Rht8* present	*r*_*1*_*r*_*1*_*R*_*8*_*R*_*8*_	116.2 ± 4.31	22.2	52	34.8	1.1	4.419	0.242
*Rht1* in heterozygous form	*R*_*1*_*r*_*1*_*r*_*8*_*r*_*8*_	112.4 ± 4.29	21.3	52	38	1.5	4.720	0.311
*Rht1* in heterozygous form + *Rht8*	*R*_*1*_*r*_*1*_*R*_*8*_*R*_*8*_	107.6 ± 3.03	22.9	55	35	1.3	4.556	0.287
*Rht1* in homozygous form	*R*_*1*_*R*_*1*_*r*_*8*_*r*_*8*_	103.7 ± 3.86	21.4	57	37	1.6	4.555	0.349
*Rht1* + *Rht8*	*R*_*1*_*R*_*1*_*R*_*8*_*R*_*8*_	90.7 ± 1.6	22.1	53	30	1.2	4.397	0.259

**Fig 4:**
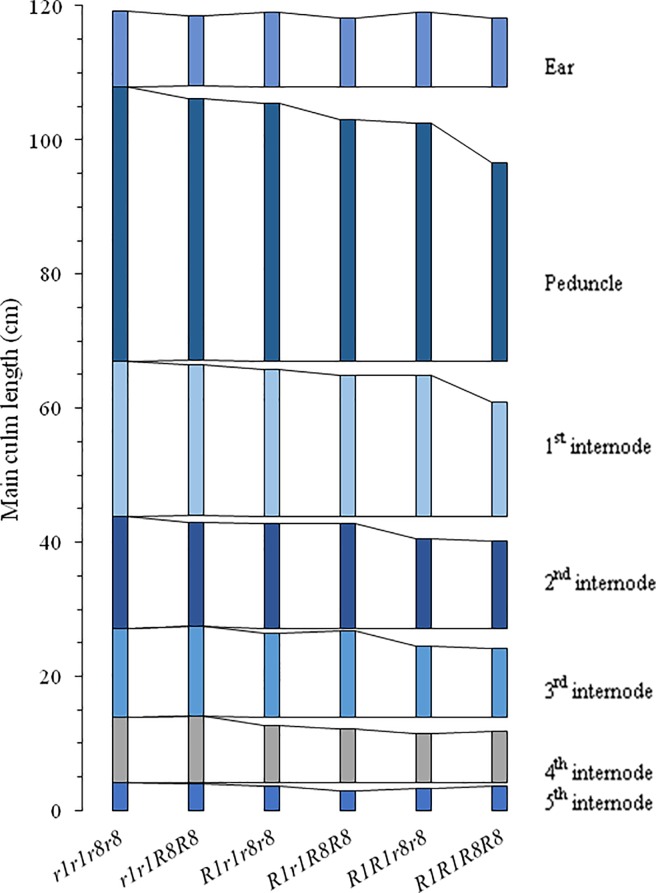
Diagrammatic representation of internodal elongation pattern in different gene combinations.

Individual internodal length was measured to know whether reduction was spread along all the internodes of wheat culm or due to a particular internode. No difference was observed in number of internodes among different genic groups. The reduction effect on wheat culm was spread along all the internodes, however, significant difference in internodal length was observed for peduncle length and first internode. As progress towards to the successive internodes lesser reduction was observed. Decrease in internodal length was larger in the lines which carry *Rht1* gene as compare to lines which carry *Rht8* gene. Maximum reduction was observed in double dwarf group carry both dwarfing genes. Difference in the internodal length reduction was observed in the group carrying *Rht1* in homozygous form and *Rht1* in heterozygous form. The schematic representation of internode elongation pattern for different genotypic classes is shown in Fig 4.

### Effect on yield and associated traits

Reduction in spike length was observed in lines with either single or double dwarfing gene, when the lines were classified into four homozygous groups. The effect on spikelet number per spike varied for different group. There was no significant difference for grain number per spike observed between the group with *Rht8* gene alone and wild type group lacking dwarfing gene. Grain number per spike was recorded maximum in the lines which carries *Rht1* gene either in heterozygous or homozygous form. No significant effect of different dwarfing genes was observed on thousand grain weight. However, slightly lower 1000-grain weight was recorded in the lines carry *Rht8* gene as compare to tall lines. Group of lines which carry only *Rht1* had comparable 1000-grain weight as that of tall group.

Highest grain yield per plot was observed in *R*_*1*_*R*_*1*_*r*_*8*_*r*_*8*_ category which carried dwarf allele of *Rht1* gene and tall allele of *Rht8* gene. It has been observed that *Rht1* caused average yield increase of 10.77% when present in heterozygous form and 18.17% when present in homozygous form whereas *Rht8* caused yield reduction of 18.27%. Decreased in plant biomass was observed in lines that carried either of the dwarfing gene or both dwarfing genes. These decrease in plant biomass and highest plot yield resulted in increased harvest index in the lines carry only *Rht1* gene (Table 2).

## Discussion

Effect of dwarfing genes on plant height and other traits were reported as highly varied with different genetic backgrounds and environmental conditions. 7–18% reduction in plant height was reported with *Rht8* gene in different studies [18, 19, 20, 21] and upto 30% of reduction in plant height with GA-insensitive genes (*Rht1* or *Rht2*) have been reported [10,18,19]. 10% reduction in plant height with *Rht8* gene [22] and 14% to 18% reduction in plant height due to *Rht8* [21]. However, in the present study relatively smaller effect (2.86%) of *Rht8* as well as of *Rht1* (13.3%) on plant height was observed. Our results also showed partial dominance behavior of *Rht1* gene as the group with *Rht1* in heterozygous form was little taller as compare to the group with *Rht1* in homozygous. Additive effect of these genes was also observed, double dwarf group were smallest among all with 24.2% reduction in plant height.

Coleoptile is the pointed protective sheath that encases the emerging shoot as it grows from the seed to the soil surface and is one of the key trait for breeding wheat for drought tolerance. Emergence ability of seedling is highly influenced by coleoptile length. For a seed to emerge successfully, seed should not be planted deeper than its coleoptile length. Reduction in coleoptile length due to dwarfing genes as observed in present study, has also been reported in several other studies. For example, 6.2% reduction in coleoptile length in the wheat cultivar containing *Rht8* gene and 25.4% reduction by *Rht1* gene was reported previously [19] which are similar the findings of present study. However, they have showed that presence of both *Rht1* and *Rht8* gene together shortened CL by 28.4% whereas in the present investigation 42.94% reduction in CL has been observed. Less effect of *Rht8* on coleoptile length and on early growth stages of wheat has been reported in many studies [10, 9, 23].

The observation of highest yield of *R*_*1*_*R*_*1*_*r*_*8*_*r*_*8*_ category is consistent with the ‘tall dwarf’ model proposed [24]. They have proposed that grain yield can be maximized by combining gibberellin-insensitive *Rht* alleles with other genes which increase both plant height and yield. Report of yield advantage with gibberellin insensitive *Rht* dwarfing genes over tall controls are also available [25]. Yield increase by *Rht1* or *Rht2* and decrease by *Rht8* have also been reported [26]. However, they showed 6.1% and 14.1% yield increase by *Rht1* and *Rht2*, respectively and 21.0% and 5.3% yield reduction by *Rht8* in two different genetic background.

The present study revealed pleiotropic effect of dwarfing genes along with effect on plant height. Under favorable conditions, lines with *Rht1* gene out yielded as compared to the lines having *Rht8* gene. However, for seedling traits lines with *Rht8* were better performing than lines with *Rht1* gene. Coleoptile length is one of the important trait for deep sowing under drought condition to capture moisture from deeper soil. For conservation agriculture, emergence under deep sowing is needed and cultivars with longer coleoptile can emerge quickly and can have better plant vigor. Hence, understanding of such pleiotropic interactions of dwarfing genes with different genetic background will facilitate effective deployment of dwarfing genes in modern varieties. The results of present study showed that lines which carry both *Rht1* and *Rht8* gene together has showed very high reduction in CL and SL. Such cultivars will not be suitable under water limiting conditions. Hence, considering the coleoptile length as an indicator for better emergence under drought conditions, it is suggested that in order to breed spring wheat cultivars for drought tolerance or water use efficiency, there is need to replace the residents dwarfing genes of Indian spring wheat cultivars which mainly consists of either *Rht1* or *Rht2*. Wheat breeders in the NWPZ of India must adopt *Rht8* as an environmentally adaptable alternative semi-dwarfing gene. The *Rht8* gene can also be successfully combined with some *Ppd* and *Vrn* genes to offer height reduction and adaptability in the niche micro environments of region so as the duration of the crop can be extended to translate into yield enhancement.

## Conclusions

Segregating population of *Rht1* and *Rht8* developed by European winter wheats and spring wheat cultivars allowed us to gain the understanding of potential of alternate dwarfing gene in Indian subcontinent. Lines with higher coleoptile length and seedling shoot length which is a deciding factor for crop establishment were identified in the study. This gave the candidate plant material to evaluate and select the material for conservation agriculture which is one of the new emerging breeding objective in light of climate change.

## Supporting information

S1 Table Mean of various agronomic traits of individual lines of different dwarfing gene combinations. (XLSX)

## References

[1] Evans LT. Crop Evolution, Adaptation and Yield. New Phytol. 1997;38: 567–574. doi: 10.2135/cropsci1998.0011183X003800010048x

[2] Worland AJ, Sayers EJ. Rht1(B. dw), an alternative allelic variant for breeding semi‐dwarf wheat varieties. Plant Breed. 1995;114: 397–400. doi: 10.1111/j.1439-0523.1995.tb00819.x

[3] Worland AJ, Sayers EJ, Korzun V. Allelic variation at the dwarfing gene Rht8 locus and its significance in international breeding programmes. Euphytica. 2001;119: 155–159.

[4] Xu XB, Zhang AM, Li XH, Sun YT. Application of dwarf resources and study advance of dwarfing genes in bread wheat. Neuc-agronomy journal. 2001;15: 188–192.

[5] Borojevic K, Borojevic K. The transfer and history of “reduced height genes” (Rht) in wheat from Japan to Europe. J Hered. 2005;96: 455–459. doi: 10.1093/jhered/esi060 15829727 10.1093/jhered/esi060

[6] Bonnett DG, Ellis M, Rebetzke GJ, Condon AG, Spielmeyer W, Richards RA Dwarfing genes in Australian wheat—present and future. In: Eastwood R et al. (Eds.), Proceedings of the 10th Australian Wheat Breeders Assembly. Mildura, 2001;154–157.

[7] Chapman SC, Mathews KL, Trethowan RM, Singh RP. Relationships between height and yield in near-isogenic spring wheats that contrast for major reduced height genes. Euphytica. 2007;157: 391–397. doi: 10.1007/s10681-006-9304-3

[8] He X, Singh PK, Dreisigacker S, Singh S, Lillemo M, Duveiller E. Dwarfing genes Rht-B1b and Rht-D1b are associated with both type i FHB susceptibility and low anther extrusion in two bread wheat populations. PLoS One. 2016;11: 1–14. doi: 10.1371/journal.pone.0162499 27606928 10.1371/journal.pone.0162499 PMC5015901

[9] Rebetzke GJ, Richards RA, Fischer VM, Mickelson BJ. Breeding long coleoptile, reduced height wheats. Euphytica. 1999;106: 159–168. doi: 10.1023/A:1003518920119

[10] Ellis MHM, Rebetzke GGJ, Chandler P, Bonnett D, Spielmeyer W, Richards RA. The effect of different height reducing genes on the early growth of wheat. Funct Plant. 2004;31: 583–589. doi: 10.1071/FP03207 10.1071/FP03207 32688930

[11] Rebetzke GJ, Ellis MH, Bonnett DG, Condon AG, Falk D, Richards RA. The Rht13 dwarfing gene reduces peduncle length and plant height to increase grain number and yield of wheat. F Crop Res. 2011;124: 323–331. doi: 10.1016/j.fcr.2011.06.022

[12] Ellis MH, Spielmeyer W, Gale KR, Rebetzke GJ, Richards RA. “Perfect” markers for the Rht-B1b and Rht-D1b dwarfing genes in wheat. Theor Appl Genet. 2002;105: 1038–1042. doi: 10.1007/s00122-002-1048-4 12582931 10.1007/s00122-002-1048-4

[13] Korzun V, Roder MS, Ganal MW, Worland AJ, Law CN. Genetic analysis of the dwarfing gene (*Rht8*) in wheat. Part I. Molecular mapping of the dwarfing gene *Rht8* on the short arm of chromosome 2D bread wheat (*Triticum aestivum* L.) Theor Appl Genet 1998;96: 1104–1109.

[14] Peng J, Richards DE, Hartley NM, Murphy GP, Devos KM, Flintham JE, et al “Green revolution” genes encode mutant gibberellins response modulators. Nature. 1999;400: 256–261. doi: 10.1038/22307 10421366 10.1038/22307

[15] Saghai-Maroof MA, Soliman KM, Jorgensen RA, Allard RW. Ribosomal DNA spacer-length polymorphisms in barley: mendelian inheritance, chromosomal location, and population dynamics. Proc Natl Acad Sci. 1984;81: 8014–8018. doi: 10.1073/pnas.81.24.8014 6096873 10.1073/pnas.81.24.8014 PMC392284

[16] Rasheed A, Wen W, Gao F, Zhai S, Jin H, Liu J, et al Development and validation of KASP assays for genes underpinning key economic traits in bread wheat. Theor Appl Genet. Springer Berlin Heidelberg; 2016;129: 1843–1860. doi: 10.1007/s00122-016-2743-x 27306516 10.1007/s00122-016-2743-x

[17] Sheoran S, Singh V, Malik R, Kundu S, Tiwari R, Kumar R, et al Distribution of dwarfing genes Rht-B1b and Rht-D1b in Indian wheat (Triticum aestivum) cultivars detected by functional markers. Indian J Agric Sci. 2013;83: 820–825.

[18] Ellis MH, Rebetzke GJ, Azanza F, Richards RA, Spielmeyer W. Molecular mapping of gibberellin-responsive dwarfing genes in bread wheat. Theor Appl Genet. 2005;111: 423–430. doi: 10.1007/s00122-005-2008-6 15968526 10.1007/s00122-005-2008-6

[19] Tang N, Jiang Y, He B Ru, Hu Y Gang. The Effects of Dwarfing Genes (Rht-B1b, Rht-D1b, and Rht8) with Different Sensitivity to GA3 on the Coleoptile Length and Plant Height of Wheat. Agric Sci China. Chinese Academy of Agricultural Sciences; 2009;8: 1028–1038. doi: 10.1016/S1671-2927(08)60310-7

[20] Rebetzke GJ, Bonnett DG, Ellis MH. Combining gibberellic acid-sensitive and insensitive dwarfing genes in breeding of higher-yielding, sesqui-dwarf wheats. F Crop Res. 2012;127: 17–25. doi: 10.1016/j.fcr.2011.11.003

[21] Wang Y, Du Y, Yang Z, Chen L, Condon AG, Hu YG. Comparing the effects of GA-responsive dwarfing genes Rht13 and Rht8 on plant height and some agronomic traits in common wheat. F Crop Res. Elsevier B.V.; 2015;179: 35–43. doi: 10.1016/j.fcr.2015.04.010

[22] Worland AJ, Korzun V, Röder MS, Ganal MW, Law CN. Genetic analysis of the dwarfing gene Rht8 in wheat. Part II. The distribution and adaptive significance of allelic variants at the Rht8 locus of wheat as revealed by microsatellite screening. Theor Appl Genet. 1998;96: 1110–1120. doi: 10.1007/s001220050846

[23] Rebetzke GJ, Richards RA. Gibberellic acid-sensitive dwarfing genes reduce plant height to increase kernel number and grain yield of wheat. Aust J Agric Res. 2000;51: 235–245. doi: 10.1071/AR99043

[24] Law CN, Snape JW, Worland AJ. The genetical relationship between height and yield in wheat. Heredity (Edinb). 1978;40: 133–151. doi: 10.1038/hdy.1978.13

[25] Flintham JE. Optimising wheat grain yield effects of rht dwarfing genes. J Agric Sci. 1997;128: 11–25.

[26] Robbins AM. Dwarfing genes in Spring wheat : an agronomic comparison of Rht-B1, Rht-D1, and Rht8. 2009; 1–63. Available: http://scholarworks.montana.edu/xmlui/bitstream/1/2139/1/RobbinsA1209.pdf

